# Mindfulness Components and Their Clinical Efficacy: A Critical Review of an Ongoing Debate

**DOI:** 10.3390/healthcare14020196

**Published:** 2026-01-13

**Authors:** Andrea Lizama-Lefno, Krystel Mojica, Mayte Serrat, Carla Olivari, Ángel Roco-Videla, Sergio V. Flores

**Affiliations:** 1 Department of Development and Postgraduate, Universidad Autónoma de Chile, Santiago 8420524, Chile; andrea.lizama@cloud.uautonoma.cl; 2 Fundación Núcleo de Investigación DOLMEN, Santiago 7580023, Chile; krystel.mojica@fundaciondolmen.org; 3 Vall d’Hebron Barcelona Hospital Campus, Vall d’Hebron Hospital Universitari, 08035 Barcelona, Spain; mayte.serrat@vallhebron.cat; 4 Escuela de Salud Pública, Universidad de Chile, Santiago 8380000, Chile; carlafob@gmail.com; 5 Facultad de Ingeniería, Universidad Católica de la Santísima Concepción, Concepción 4090541, Chile; aroco@ucsc.cl; 6 Centro de Investigación en Medicina de Altura, Universidad Arturo Prat, Iquique 1110939, Chile; 7 Facultad de Ciencias de la Salud, Universidad Católica Silva Henríquez, Santiago 8280354, Chile

**Keywords:** mindfulness, clinical efficacy, meditation, cognitive behavioral therapy, mental health, health education

## Abstract

**Highlights:**

**What is the main findings?**

- Mindfulness-based interventions show moderate clinical efficacy, with outcomes highly dependent on their specific components (meditation, psychoeducation, and informal practice). Future research must conduct rigorous head-to-head and longitudinal studies comparing mindfulness-based interventions with other active treatments, to clarify which elements sustain long-term clinical outcomes and to avoid overstated claims.

**What are the implication of the main findings?**

- Clinical application of mindfulness should be guided by insights from component analyses, rather than by the assumption that meditation alone constitutes the primary therapeutic mechanism.
- Future research must conduct comparative and longitudinal studies to clarify which elements sustain long-term clinical outcomes and to avoid overstated claims.

**Abstract:**

The rapid expansion of mindfulness research has generated both enthusiasm and controversy regarding its actual clinical value. While meditation is often regarded as the central mechanism of mindfulness-based interventions, other components such as psychoeducation and informal practice may play an equally significant role in improving mental health outcomes. This critical review examines the relative contributions of these elements to the therapeutic impact of mindfulness and clarifies the extent to which its effects are comparable to established treatments, particularly Cognitive Behavioral Therapy (CBT). Evidence from meta-analyses and high-quality trials indicates that mindfulness programs achieve moderate efficacy in reducing symptoms of anxiety, depression, and stress, but effect sizes are frequently inflated by methodological limitations. Importantly, cognitive and emotional regulation skills, especially acceptance and non-judgment, appear to sustain long-term benefits more consistently than meditation alone. These findings highlight the need for rigorous longitudinal studies and component-focused designs to identify the mechanisms that drive clinical change. By distinguishing between evidence-based applications and overstated claims, this review contributes to a more balanced understanding of mindfulness and its appropriate integration into healthcare.

## 1. Introduction

Cognitive Behavioral Therapy (CBT) and Mindfulness-Based Therapies (MBTs) represent two influential approaches in modern psychotherapy, each with distinct theoretical foundations and clinical applications. While CBT has solidified its position as one of the most effective therapies for treating a variety of mental disorders, MBTs have gained increasing recognition due to their focus on meditation and acceptance as a therapeutic strategy, with a growing number of scientific publications.

Cognitive Behavioral Therapy (CBT) is categorized as a second-generation psychological therapy, emphasizing the identification and modification of harmful automatic thoughts and maladaptive behavioral patterns that contribute to psychological distress [1,2]. This approach is based on the premise that negative thought patterns and deeply ingrained core beliefs can shape the perception of reality, leading to emotional distress and maladaptive behaviors. By employing cognitive restructuring and behavioral interventions, CBT aims to promote more adaptive cognitive and emotional processing, ultimately enhancing psychological well-being [3]. While CBT has been extensively validated across a range of clinical conditions, contemporary approaches have expanded beyond strict cognitive restructuring, integrating elements of mindfulness, acceptance, and psychological flexibility to enhance treatment outcomes [4].

Mindfulness-Based Therapies (MBTs) are a set of interventions that integrate meditative practices within psychotherapy, emphasizing acceptance, metacognition, and cognitive flexibility. Among the most established MBTs are Mindfulness-Based Stress Reduction (MBSR) [5] and Mindfulness-Based Cognitive Therapy (MBCT) [6]. Additionally, some third-wave cognitive–behavioral therapies, such as Acceptance and Commitment Therapy (ACT) [7,8,9] and Dialectical Behavior Therapy (DBT) [10,11], incorporate mindfulness techniques within broader clinical frameworks. Although these interventions differ in their theoretical foundations, they all utilize mindfulness as a strategy to cultivate present-moment awareness and reduce automatic reactivity to thoughts and emotions [12,13].

Unlike CBT, which traditionally emphasizes modifying thought content, MBTs foster cognitive flexibility by encouraging individuals to observe their internal experiences without automatically reacting to them. Rather than directly challenging maladaptive thoughts, MBTs aim to alter how individuals relate to them, promoting a stance of openness and acceptance [14,15]. This approach has demonstrated effectiveness in reducing stress and anxiety, as well as in preventing relapse in depression, particularly in clinical populations where rumination and emotional dysregulation play a significant role [16,17,18,19].

Mindfulness, originally rooted in Buddhist meditative traditions, was adapted into clinical psychology through Jon Kabat-Zinn’s development of Mindfulness-Based Stress Reduction (MBSR) in the 1970s. While its integration into Western psychotherapy has prioritized scientific validation, this process has also led to the selective appropriation of mindfulness principles, often stripping them of their original ethical and philosophical dimensions [20,21]. This shift has facilitated its widespread application in healthcare and mental health settings, yet it has also raised concerns regarding its commodification and dilution in mainstream wellness culture. Understanding this duality is crucial to critically evaluating both its legitimate therapeutic benefits and the risks of its misrepresentation in popular discourse.

MBSR is widely used for managing anxiety, stress, and preventing depression relapse. While evidence supports its efficacy, effects are moderate and vary by population and study design [18,22]. In chronic pain and inflammatory conditions, benefits appear to be more related to psychological adaptation than direct symptom relief, highlighting the need for a critical evaluation of its clinical applications [23].

Although studies of mindfulness-based therapies (MBTs) have documented their benefits, the magnitude and consistency of these effects vary across conditions, populations, and study designs, warranting cautious interpretation. Research has consistently indicated that the impact of mindfulness-based interventions is often overstated, particularly when compared with well-established treatments such as cognitive–behavioral therapy [24]. Additionally, when assessing mindfulness-based interventions, it remains unclear which specific components drive therapeutic outcomes and how these interact with other psychological processes [25,26].

This article analyzes the key components integrated into standardized mindfulness programs—meditative, psychoeducational, and conceptual elements, as well as CBT strategies—to clarify their individual and combined contributions to clinical efficacy and well-being. A critical analysis of the psychological mechanisms underlying the benefits of mindfulness practice is necessary to differentiate its short-term effects from its potential long-term benefits and to prevent misinterpretations that could contribute to its commercial overstatement.

Here, clinical efficacy is understood as the measurable improvement in mental health outcomes, including symptom reduction, relapse prevention, and functional recovery, as commonly evaluated in randomized controlled trials and meta-analyses. In this review, the term “meditation” is used in its clinical and operational sense, referring to structured attentional and awareness-based practices as defined in standardized mindfulness-based interventions (e.g., MBSR, MBCT), rather than in its traditional or spiritual meaning within ancient contemplative traditions.

The aim of this article is to promote a critical and evidence-based approach to assessing the benefits of mindfulness practice, fostering academic and professional debate on its real advantages and limitations. By distinguishing between well-supported applications and overstated claims, this perspective seeks to reinforce the legitimacy of mindfulness as a therapeutic tool while addressing concerns about its commodification and the proliferation of unrealistic expectations.

This article follows a critical narrative review methodology. Rather than conducting a systematic review with predefined protocols, risk-of-bias assessments, or exhaustive literature screening, the objective is to synthesize and critically interpret high-quality empirical and conceptual contributions relevant to the clinical efficacy of mindfulness components. The search strategy was iterative and concept-driven, prioritizing meta-analyses, systematic reviews, and randomized controlled trials that inform current debates on mechanism, clinical value, and methodological limitations. This approach is consistent with the purpose of the manuscript: to offer an integrative and critical evaluation of how meditative, psychoeducational, and cognitive–behavioral elements contribute to therapeutic outcomes.

## 2. Conceptualization of the Term Mindfulness

The term mindfulness originates from the Pali word Sati, which translates as “attention” or “awareness” [27]. In Buddhist doctrine, Sati is described as a form of skillful attention, constituting the seventh factor of the Noble Eightfold Path, known as Samma-sati in Pali scriptures [28].

Although there is a broad academic consensus on defining mindfulness as a heightened state of awareness focused on present-moment experience, its conceptualization varies across disciplines. Some perspectives emphasize its cognitive and attentional mechanisms [29,30], while others highlight its ethical and contemplative dimensions rooted in Buddhist traditions [15,31]. The Spanish translation “Atención Plena” captures the core idea of sustained, non-judgmental awareness, though its meaning may shift depending on whether it is framed as a clinical intervention or a spiritual practice.

A more pragmatic conceptualization of mindfulness defines it as a universal human capacity for moment-to-moment awareness of mental content [32,33]. While mindfulness is often described as an accessible and familiar experience, equating it with a purely spontaneous state may oversimplify its mechanisms and therapeutic applications. Although commonly associated with meditation, mindfulness and meditation are not interchangeable terms. Mindfulness meditation encompasses a wide range of practices with varying objectives, whereas mindfulness itself can be conceptualized as a cognitive state, a cultivated disposition, or an intentional practice. While meditation may enhance mindfulness, Brown and Ryan [30] highlight that mindfulness can also arise independently through attentional training and self-regulation processes.

In this review, we adopt operational definitions to ensure conceptual precision. Mindfulness is defined as a metacognitive process involving sustained, non-judgmental awareness of present-moment experience [29]. Meditation refers to formal practices designed to cultivate such awareness through focused attention or open monitoring [15]. Psychoeducation is understood as the structured transmission of cognitive–behavioral information that supports emotional regulation and self-management [1]. These distinctions clarify the scope of each construct and will be used consistently throughout the manuscript.

In this review, we adopt operational definitions grounded in empirical research to ensure conceptual precision. Mindfulness is defined as a metacognitive process of sustained, non-judgmental awareness of present-moment experience [29], and in studies it is typically measured through validated instruments such as the MAAS or FFMQ. Meditation refers to the formal practices designed to cultivate this awareness—most commonly focused-attention or open-monitoring exercises [15]—and is evaluated through prescribed session duration, frequency, and adherence. Psychoeducation is understood as the structured transmission of cognitive–behavioral information that supports emotional regulation and self-management [1], and its effects are commonly assessed through changes in understanding, skills, and engagement. These distinctions clarify how each construct functions empirically within mindfulness-based interventions and will be used consistently throughout the manuscript.

In contemporary Western societies, the increasing demand for well-being and relief from suffering has contributed to the widespread diffusion and commercialization of interventions that promise these outcomes, including medical, psychological, and contemplative approaches. This phenomenon extends across medical and psychological treatments, pharmaceutical solutions, alternative therapies, and self-help models that integrate contemplation, relaxation, and meditation. The perceived legitimacy of these approaches is often reinforced when backed by scientific evidence, making them focal points of research in medicine, neurophysiology, and psychology [34]. While the perceived legitimacy of these approaches is often reinforced by scientific evidence, the growing demand for such interventions raises questions about whether their promotion is primarily grounded in empirical validation or influenced by broader market dynamics.

The distinction between mindfulness and traditional meditative practices has led to a consensus identifying two core components. The first is attention regulation, which involves deliberately directing perception to specific aspects of present experience while inhibiting elaborative cognitive processing. This means reducing the tendency to fixate on thoughts, emotions, or bodily sensations such as pain, or to become distracted by them. Instead, these experiences are to be recognized, acknowledged, and observed without immediate reaction before allowing them to dissipate [35]. This process is said to foster meta-awareness—the capacity to recognize one’s own mental activity as transient rather than absolute [29].

Both attention and awareness are fundamental to consciousness, enabling individuals to notice stimuli without becoming cognitively overwhelmed by them. Bishop et al. [29] describe this as a form of attentional self-regulation, which involves consciously redirecting focus—such as returning to the breath—when a sensation, thought, or emotion arises. This mechanism reduces the likelihood of engaging in maladaptive rumination or mind-wandering. Rather than merely suppressing cognitive processing, this approach facilitates cognitive flexibility, allowing individuals to maintain awareness of stimuli while disengaging from automatic or habitual reactions.

A second core component of mindfulness is the attitudinal stance with which one engages with present-moment experiences. This mindset is often described as a combination of curiosity, openness, and acceptance [29,36]. However, acceptance in this context does not imply passive resignation or suppression of discomfort, but rather an intentional acknowledgment and non-reactive engagement with thoughts, emotions, and sensations [30]. Kabat-Zinn [15] proposed that mindfulness practice fosters seven attitudinal factors: non-judgment, observing experiences without rigid categorization; patience, allowing events to unfold naturally; beginner’s mind, approaching experiences with curiosity; trust, developing confidence in one’s ability to remain present; non-striving, letting go of the need to control experiences; acceptance, recognizing reality without avoidance; and letting go, releasing attachments and expectations. While these principles serve as practical guidelines for mindfulness cultivation, their application in clinical and research settings remains a topic of debate, particularly regarding their necessity for achieving therapeutic benefits.

Since mindfulness, influenced by Buddhist philosophy, is traditionally associated with the cessation of suffering, the principles of acceptance and non-judgment are particularly emphasized. Several authors argue that psychological distress is exacerbated by resistance to experiences, and that fostering acceptance and non-judgment can help reduce suffering by diminishing the habitual tendency to avoid or suppress unpleasant emotions and sensations. However, the extent to which these principles translate into measurable clinical benefits remains a subject of debate, as empirical evidence varies regarding their role as distinct therapeutic mechanisms.

## 3. Protocolization of Mindfulness and Its Scientific Evidence

The protocolization of mindfulness involves the development of structured formats for its instruction and practice across different contexts, particularly in clinical, therapeutic, and wellness settings. Standardized protocols aim to establish specific session structures, durations, and implementation procedures to facilitate scientific evaluation and ensure consistency in application. This framework has contributed to the empirical validation of mindfulness-based interventions; however, it also raises concerns regarding the extent to which rigid standardization may overlook the individualized and context-dependent nature of mindfulness practice [37,38].

Mindfulness-based intervention protocols emphasize the cultivation of present-moment awareness with intentionality and non-judgment. Sessions typically involve guided exercises focusing on breathing, bodily sensations, thoughts, and emotions. Techniques such as body scans and mindful breathing are commonly used to enhance attentional control and emotional regulation. A key objective of these protocols is to develop the ability to observe mental and emotional processes without over-identifying with them or reacting automatically. This capacity allows individuals to recognize maladaptive cognitive and emotional patterns, although the extent to which this leads to measurable clinical benefits varies across populations and study designs.

A fundamental aspect of mindfulness-based intervention protocols is the incorporation of daily practice, both formal and informal. Formal practice involves structured meditation exercises, often guided by recordings or specific instructions. Informal practice, in contrast, integrates mindfulness into daily activities through attentional and cognitive training strategies. While adherence to these practices is considered crucial for maximizing potential benefits [15,26], research indicates significant variability in engagement levels and outcomes, raising questions about the extent to which daily practice is necessary for achieving meaningful therapeutic effects.

Mindfulness-Based Therapy (MBT) programs are structured, evidence-informed interventions, with Mindfulness-Based Stress Reduction (MBSR) and Mindfulness-Based Cognitive Therapy (MBCT) receiving the strongest empirical support. However, the degree of standardization and the influence of intervention duration remain subjects of ongoing research. While shorter programs (six weeks) tend to yield smaller effect sizes compared to eight-week interventions, particularly in reducing stress and anxiety [34,39,40], evidence regarding the necessity of extended programs is mixed. Longer interventions (10–12 weeks) have shown greater efficacy in populations with severe psychological conditions or in facilitating long-term behavioral changes [41], yet follow-up studies beyond 12 weeks remain scarce, limiting conclusions about the sustainability of effects over time [42].

While mindfulness-based interventions show short-term benefits, systematic reviews and meta-analyses highlight the need for more rigorous research to establish their long-term efficacy. Current findings suggest that while these interventions can reduce stress and improve emotional regulation, the durability of these effects remains uncertain [18,43]. Well-controlled studies with larger samples are required to determine the influence of factors such as intervention duration, practice frequency, and individual differences. Future research should also address methodological inconsistencies and assess whether long-term benefits extend beyond symptom reduction to broader psychological and behavioral changes [18,43,44,45,46,47].

The effectiveness of mindfulness-based interventions is influenced by multiple factors, including the time dedicated to formal and informal practice between sessions [48], the severity of symptoms at baseline [49], and the facilitator’s level of expertise in delivering the program [50,51]. While these elements are often assumed to enhance outcomes, their specific impact remains unclear, as studies show variability in adherence rates, symptom progression, and facilitator effects across different populations.

Moreover, complementing these findings with qualitative approaches emerges as a valuable alternative to enrich the understanding of the experiential impact from the participants’ perspective, including perceived benefits, practice challenges, difficulties, perceptions of personal growth, awareness—i.e., insights about themselves that they have become aware of—and finally, gathering recommendations from the narratives, thereby significantly expanding and strengthening the body of knowledge in this regard [52,53,54].

## 4. Proliferation of Scientific Evidence

The increasing scientific interest in mindfulness raises the question of why maintaining sustained attention to internal and external experiences has become a focal point of research. Reflecting this trend, the number of mindfulness-related publications has grown exponentially. Between 1966 and 2021, 16,581 publications were indexed in the Web of Science, including 14,682 articles and 1899 reviews that referenced mindfulness in the title, abstract, or keywords. Bibliometric analyses also show a surge in international research collaborations, particularly among institutions in North America and Europe, with the United States, England, Australia, Canada, Germany, and Switzerland producing the highest volume of publications [55]. However, the most frequently cited research today originates from countries such as Turkey, China, Peru, Vietnam, and Pakistan, highlighting a shift in global scientific contributions and a growing interest in perspectives beyond the Western framework.

**Figure 1:**
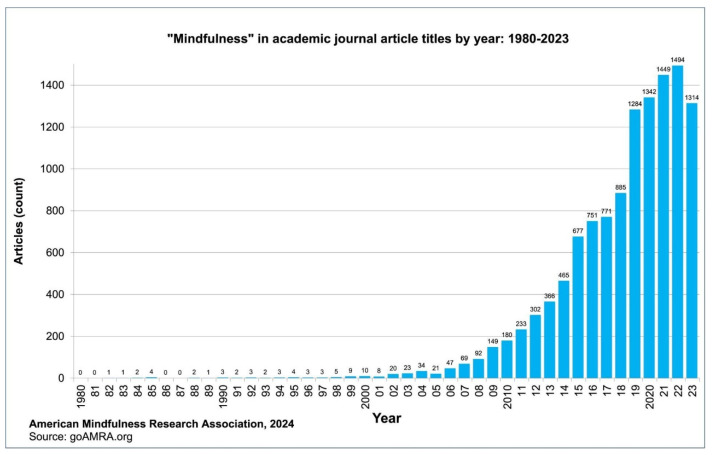
Journal articles with the title “Mindfulness” published per year: 1980–2023. Source: American Mindfulness Research Association [56].

According to the American Mindfulness Research Association [56], the number of published articles grew from 10 in 2000 to 1362 in 2021 (Figure 1).

Scientific inquiry has sought to understand the historical significance of mindfulness and its persistence across cultures. Contemporary research continues to explore its applications, expanding the evidence for mindfulness-based interventions while adapting them to diverse contexts and populations with varying psychological and physiological needs. However, the extent to which these adaptations maintain the integrity of traditional mindfulness practices or enhance their clinical efficacy remains a subject of debate.

A search in Google Scholar indicates a continuous rise in publications referencing “mindfulness” [57]. Similarly, PubMed, a database specializing in medical literature, lists over thirty thousand entries under the same term, including 3900 publications from 2024. Of these, 9.2% are randomized controlled trials (RCTs), 7.9% are systematic reviews, and 3.4% are meta-analyses [58]. While these figures reflect the growing academic interest in mindfulness, they also underscore the need for critical evaluation, as increased publication volume does not necessarily equate to higher-quality evidence or conclusive findings.

Ferreira & Demarzo [59] report an annual growth rate of 11.8% in mindfulness-related publications between 1999 and 2019, with a peak in 2019. The increasing body of research on mindfulness has contributed to its widespread adoption in Western contexts, extending beyond clinical applications into areas such as education, sports, corporate settings, and personal development [10]. While mindfulness-based interventions have demonstrated benefits for mental and physical health, as well as cognitive and emotional regulation, their growing popularity has also led to concerns regarding the extent to which their application aligns with evidence-based practice. The use of mindfulness as a generalized tool for well-being and performance enhancement raises questions about its actual effectiveness in these diverse domains and the risk of oversimplification in its implementation.

## 5. The Revolution of Moderate Efficacy: A Dilemma Between Quantity and Quality

Several authors have critically examined the ideological framing of scientific evidence, questioning both the magnitude of mindfulness’s effects and the excessive enthusiasm surrounding its clinical and social applications, given that its reported benefits are often mild to moderate [24,39,40,60]. Errasti-Pérez et al. [60] argue that although mindfulness researchers frequently caution against viewing mindfulness as a panacea, their discourse and practices sometimes contradict this stance. Terms such as “mindful eating” [61] and “mindful sex” [62] illustrate how mindfulness has been extended beyond its core applications, often in ways that appear driven more by commercial appeal than by empirical support. This trend reflects a broader tendency in modern Western societies to secularize, commodify, and rebrand elements of Eastern traditions for mass consumption [63].

The increasing popularity of mindfulness in Western societies has generated enthusiasm that often surpasses the strength of the scientific evidence supporting its benefits. A significant portion of the literature faces methodological limitations, with some studies potentially shaped by the commercial appeal of mindfulness rather than by rigorous empirical inquiry [24,64]. Farias and Wikholm [65] argue that the secularization and commodification of mindfulness have diluted its conceptual depth, transforming it into a product that aligns more with market demands than with a genuine commitment to human well-being. These critiques highlight the need for a critical reassessment of how mindfulness is researched and implemented, given the lack of standardized protocols and the tendency to generalize outcomes across diverse populations. Ensuring that its continued expansion is driven by scientific integrity rather than commercial opportunism remains essential.

In the literature on the efficacy of mindfulness-based therapies (MBTs) for anxiety and depression, meta-analyses provide a more nuanced perspective, particularly when comparing MBTs with other therapeutic approaches. The following paragraphs highlight key findings from several studies and meta-analyses, showcasing both the effectiveness and the limitations of MBTs.

The meta-analysis conducted by Hofmann et al. [10] is one of the most frequently cited studies on the efficacy of mindfulness-based therapies (MBTs). This research found that these therapies have a moderate effect in reducing symptoms of anxiety and depression, with effect sizes of 0.63 for anxiety and 0.59 for depression. However, it is important to note that these effects were primarily observed in comparison to inactive controls, which may overestimate the true effectiveness of mindfulness. When compared to other active treatments, the relative efficacy of MBTs dropped significantly, with effect sizes of 0.22 for anxiety and 0.23 for depression, indicating a small effect.

Khoury et al. [39,40] also found that MBTs have a moderate effect in alleviating symptoms of anxiety and depression, with the most significant improvements observed when compared to usual care, placebos, or inactive controls. However, their effectiveness appears less robust when evaluated against well-established interventions such as Cognitive Behavioral Therapy (CBT), which has a more extensive evidence base. In some cases, MBTs did not demonstrate clear advantages overactive treatments, and direct comparisons were not always conducted, limiting definitive conclusions about their relative efficacy.

Mindfulness-Based Cognitive Therapy (MBCT) has been widely studied, largely due to its foundation in Cognitive Behavioral Therapy (CBT), one of the most well-established psychotherapies. While emerging evidence suggests its potential benefits, several studies caution that its efficacy remains inconclusive, partly due to the limited number of high-quality randomized trials [66]. Coelho et al. [67] highlight methodological challenges in clinical trials, noting that observed improvements cannot be definitively attributed to specific effects of MBCT. In a study involving individuals with a history of childhood trauma, MBCT significantly reduced relapse rates but did not show a clear advantage over an active control treatment [68]. Overall, there is broad agreement among researchers that further investigation is needed to determine whether MBCT provides distinct therapeutic benefits beyond those of other interventions.

In summary, while mindfulness-based programs have demonstrated effectiveness, their impact is generally moderate and more pronounced when compared to inactive controls. However, when evaluated against therapies with stronger empirical support, such as CBT, their relative efficacy is notably lower. This calls for measured enthusiasm and a rigorous interpretation of the available evidence to avoid overstating their benefits. This trend has been consistently reported across both seminal and recent reviews, which converge in describing mindfulness-based interventions as producing moderate, heterogeneous, and mostly short-term effects across anxiety, depression, and chronic pain outcomes [12,16,17,44,45]. Although research findings suggest potential advantages, it is essential to maintain a critical and methodical approach when assessing their actual clinical relevance and long-term impact.

## 6. Discussion

### 6.1. Which Component Contributes Most to Mindfulness’s Benefits for Mental Health?

Mindfulness meditation is often regarded as the central component of mindfulness programs, yet its role should be critically examined in relation to other key elements, such as psychoeducation and informal practice, which are traditionally integrated into cognitive–behavioral therapies. While some studies highlight meditation as a primary mechanism for short-term reductions in stress and anxiety, others suggest that cognitive and behavioral learning processes—particularly the cultivation of acceptance and non-judgment—may play an equally or even more significant role in sustaining long-term benefits [26]. This raises important questions about whether meditation alone is sufficient to drive mindfulness-related improvements or if its effects are dependent on complementary psychoeducational and cognitive strategies.

Beyond their individual effects, these components likely interact dynamically within mindfulness-based interventions. Meditative practice can enhance attentional control and emotional awareness, while psychoeducational elements provide cognitive frameworks that facilitate understanding and integration of these experiences into daily life. Informal practice, in turn, reinforces behavioral consistency and the transfer of learned skills beyond structured sessions. Such reciprocal influences suggest a synergistic relationship rather than independent effects.

High-quality studies—those with low risk of bias, rigorous randomization, and adequate control conditions—tend to report smaller effect sizes for mindfulness meditation compared to studies with methodological limitations [18,24,69]. This suggests that the benefits of it may have been overestimated in less robust research. However, even in well-controlled trials, meditation has shown moderate effects in reducing stress and anxiety, highlighting its potential therapeutic value while reinforcing the need for continued scrutiny of its long-term impact and mechanisms of action.

The cultural adaptability of mindfulness-based interventions is another key consideration. While mindfulness originated in Eastern contemplative traditions emphasizing ethical conduct and collective well-being, its integration into Western clinical practice has often focused on individual symptom relief and performance enhancement. This cultural recontextualization may influence both the meaning and perceived benefits of practice, potentially shaping engagement, adherence, and outcomes. Recognizing these cultural differences is essential for developing interventions that are not only evidence-based but also contextually sensitive and globally relevant.

Meta-analyses examining the impact of meditation, particularly Transcendental Meditation (TM), suggest that TM may reduce symptoms of anxiety and depression, with greater effects observed in individuals with high baseline anxiety [18,70]. Some findings indicate that TM outperforms techniques like progressive relaxation or other meditation forms. However, it is critical to acknowledge that these studies do not directly compare TM with Cognitive Behavioral Therapy (CBT), which remains the gold standard for anxiety treatment. Additionally, many of these studies are dated and present methodological limitations, raising concerns about their generalizability to current clinical settings. These factors underscore the need for more recent, high-quality trials to validate the purported benefits of TM [69].

Research on other meditative practices also suggests potential benefits in reducing anxiety and depression [44]. However, these studies are fewer in number and often methodologically limited, making it difficult to draw definitive conclusions. The reported effects are generally comparable to those observed with TM, though they vary depending on the technique used and the population studied. Additionally, long-term effects remain largely unexplored, as most available research focuses on short- and medium-term interventions—with short-term studies typically lasting up to 8 weeks, medium-term between 3 and 6 months, and long-term those exceeding 12 months—with limited follow-up periods. This lack of extended studies raises uncertainties about whether continuous meditation practice is necessary to sustain its benefits. Given the scarcity of specific studies and the variability in methodological approaches, current conclusions should be interpreted with caution.

An important area for further research is the degree to which improvements observed in mindfulness interventions stem from meditation practice versus the acquisition of cognitive and emotional regulation skills, such as acceptance and non-judgment—key learning outcomes in Mindfulness-Based Cognitive Therapy (MBCT). Disentangling these contributing factors is crucial to identifying which components drive the most substantial therapeutic effects. It is important to clarify that the effectiveness of mindfulness-based cognitive therapy (MBCT) does not depend on a high level of emotional intelligence (EQ). Instead, emotional awareness and regulation—skills that MBCT itself seeks to cultivate—may act as mediating factors that enhance treatment outcomes without constituting a prerequisite for therapeutic success.

Additionally, longitudinal studies are necessary to assess not only the duration of these effects but also the distinct impact of different mindfulness-based therapy components on mental health outcomes. Specifically, future research should clarify which elements are most effective for alleviating short-term distress and which contribute to sustained psychological well-being over time.

The impact of mindfulness meditation in short-term programs, such as the widely used eight-week protocols, requires further examination, particularly given that research suggests its most definitive effects emerge over the long term [71,72]. Although Transcendental Meditation and other mindfulness-based practices have demonstrated some effectiveness in reducing anxiety and depression, head-to-head comparisons with Cognitive Behavioral Therapy (CBT) have been relatively scarce. However, a recent meta-analysis synthesizing 30 randomized controlled trials [73] found MBT and CBT to be statistically equivalent in treating current adult depression. This updated evidence refines our previous statement, indicating that while comparative studies are increasing, their scope and long-term follow-up data remain limited, preventing firm conclusions about relative mechanisms or durability of effects.

Beyond these empirical uncertainties, it is important to note how the present review advances previous critical syntheses in the field. While influential works such as Van Dam et al. [24] emphasized methodological limitations in mindfulness research as a whole, and Creswell [43] summarized broad intervention outcomes, the current review offers a distinct contribution by examining the relative clinical impact of specific components within mindfulness-based programs. By contrasting meditative, psychoeducational, and cognitive–behavioral elements, this review provides a component-level perspective that helps clarify which mechanisms are most strongly supported by empirical evidence and where conceptual assumptions may have overstated the role of meditation.

### 6.2. Is Formal Meditation Indispensable?

A critical question is whether the exclusion of formal meditation would substantially diminish the effectiveness of mindfulness-based interventions. While meditation is frequently considered the foundation of these programs, some researchers hypothesize that its benefits may stem primarily from psychoeducational and informal practice components, which align closely with principles from cognitive–behavioral therapy. The widespread commercialization of mindfulness and its rapid expansion in Western societies have raised concerns that the emphasis on meditation may be influenced more by market dynamics than by solid empirical evidence of its actual clinical efficacy.

As mindfulness practice continues to expand, research must move beyond short-term, standardized interventions. There is a notable lack of long-term follow-up studies comparing health outcomes between individuals who engage in meditation within structured programs and those who integrate it into their daily lives. It remains unclear whether the benefits of meditation are enduring or if they diminish once structured practice ends. A fundamental question is whether mindfulness-based interventions consistently lead to sustained health improvements in Western societies or if their effects are primarily transient, addressing temporary discomfort rather than producing lasting change.

Recent studies provide valuable insights into this issue: Trapani et al. [74] demonstrated that mindfulness-based interventions initiated during pregnancy have measurable long-term benefits on postpartum depression, while Ji et al. [75] showed that mindfulness mediates well-being through stable psychological and physiological processes in adolescents. Complementing these findings, Liu et al. [76] reported that an eight-week MBSR program for ICU nurses maintained significant effects twelve weeks after completion, indicating that mindfulness training can yield enduring benefits across different populations and contexts. Similarly, Zhang et al. [77] found that, although mindfulness-based approaches remain clinically valuable, their efficacy is moderate compared to other non-pharmacological interventions, reinforcing the need for nuanced evaluation of their long-term therapeutic potential.

Integrating these perspectives, mindfulness-based interventions can be conceptually understood as operating through three complementary mechanisms: (a) attentional regulation cultivated by meditation, which enhances present-moment awareness; (b) cognitive restructuring facilitated by psychoeducation, which supports reappraisal and emotional understanding; and (c) behavioral integration achieved through informal practice, which consolidates learned skills into daily life.

The critical challenge is to ensure that scientific knowledge on mindfulness is effectively translated into interventions that genuinely improve public health, rather than serving commercial interests or the instrumentalization of research for profit. Addressing these concerns is essential to clarifying the true scope of mindfulness as a clinical, social, cultural, and even political tool, while also engaging with critiques that argue it has become a commodified phenomenon detached from its original intent—enhancing well-being and quality of life.

## 7. Conclusions

The primary challenge in mindfulness-based interventions is determining which aspects are responsible for contributing to their effectiveness, and what mechanisms may be at work. While meditative practice, often associated with mindfulness training and stress reduction, has demonstrated benefits in alleviating symptoms of anxiety and depression, a critical question remains: is meditation alone sufficient to sustain these effects over time, or are additional cognitive, emotional, and behavioral factors necessary? Understanding whether the long-term benefits of mindfulness stem from mindfulness meditation alone, or also from additional contributions from complementary psychological processes, is essential for refining its clinical applications and preventing its overgeneralization as a universal remedy.

Recent high-quality evidence continues to support the moderate but consistent clinical efficacy of MBIs across populations and settings. However, these benefits appear to depend not only on meditation but also on psychoeducational and cognitive–behavioral elements that promote acceptance, non-judgment, and emotional regulation. Such skills have shown comparable or even greater contributions to long-term mental health outcomes than formal meditation practice alone.

Although mindfulness-based interventions have demonstrated effectiveness in reducing anxiety and depression, direct comparisons with Cognitive Behavioral Therapy (CBT) remain limited. Evidence from recent meta-analyses suggests that both approaches may yield comparable therapeutic outcomes, possibly through shared mechanisms involving cognitive restructuring and self-regulation. In contrast, findings on Transcendental Meditation (TM) are more heterogeneous and should be interpreted with caution, as TM differs conceptually and methodologically from mindfulness-based approaches. Nonetheless, further well-controlled, longitudinal research is required to clarify whether sustained meditation practice provides additional or distinct long-term advantages.

The psychoeducational components of mindfulness, which encourage present-moment awareness and a non-judgmental attitude, may play a significant role in reducing rumination—a core mechanism underlying anxiety and depression. This raises the question of whether long-term symptom improvement is primarily driven by the internalization of these cognitive and emotional strategies rather than by meditation itself.

Overall, mindfulness-based interventions show moderate clinical efficacy, with outcomes highly dependent on their specific components—meditation, psychoeducation, and informal practice. Cognitive and emotional regulation skills such as acceptance and non-judgment may be the most critical drivers of long-term well-being. Identifying how these elements interact to sustain psychological benefits is key for optimizing intervention design and ensuring mindfulness remains a scientifically grounded and contextually adaptable therapeutic tool.

## Data Availability

No new data were created or analyzed in this study. Data sharing is not applicable to this article.

## Abbreviations

The following abbreviations are used in this manuscript:

AMRA	American Mindfulness Research Association
APA	American Psychological Association
APC	Article Processing Charge
CBT	Cognitive Behavioral Therapy
DBT	Dialectical Behavior Therapy
MBCT	Mindfulness-Based Cognitive Therapy
MBSR	Mindfulness-Based Stress Reduction
